# Causal relationship between rheumatoid arthritis and hypothyroidism or hyperthyroidism: a bidirectional two-sample univariable and multivariable Mendelian randomization study

**DOI:** 10.3389/fendo.2023.1256208

**Published:** 2023-11-29

**Authors:** Rui Lai, Xinmin Deng, Xiaofeng Lv, Qian Liu, Kun Zhou, Dezhong Peng

**Affiliations:** ^1^ School of Acupuncture and Tuina, Chengdu University of Traditional Chinese Medicine, Chengdu, China; ^2^ School of Clinical Medicine, Chengdu University of Traditional Chinese Medicine, Chengdu, China; ^3^ The Third Clinical School of Zhejiang Chinese Medicine University, Hangzhou, China

**Keywords:** Mendelian randomization, rheumatoid arthritis, hypothyroidism, hyperthyroidism, causal relationship

## Abstract

**Objective:**

The causal relationship between Rheumatoid arthritis (RA) and hypothyroidism/hyperthyroidism remains controversial due to the limitations of conventional observational research, such as confounding variables and reverse causality. We aimed to examine the potential causal relationship between RA and hypothyroidism/hyperthyroidism using Mendelian randomization (MR).

**Method:**

We conducted a bidirectional two-sample univariable analysis to investigate the potential causal relationship between hypothyroidism/hyperthyroidism and RA. Furthermore, we performed a multivariate analysis to account for the impact of body mass index (BMI), smoking quantity, and alcohol intake frequency.

**Results:**

The univariable analysis indicated that RA has a causative influence on hypothyroidism (odds ratio [OR]=1.07, 95% confidence interval [CI]=1.01–1.14, P=0.02) and hyperthyroidism (OR=1.32, 95% CI=1.15–1.52, P<0.001). When hypothyroidism/hyperthyroidism was considered as an exposure variable, we only observed a causal relationship between hypothyroidism (OR=1.21, 95% CI=1.05–1.40, P=0.01) and RA, whereas no such connection was found between hyperthyroidism (OR=0.91, 95% CI=0.83–1.01, P=0.07) and RA. In the multivariate MR analyses, after separately and jointly adjusting for the effects of daily smoking quantity, alcohol intake frequency, and BMI, the causal impact of RA on hypothyroidism/hyperthyroidism and hypothyroidism on RA remained robust. However, there is no evidence to suggest a causal effect of hyperthyroidism on the risk of RA (P >0.05).

**Conclusion:**

Univariate and multivariate MR analyses have validated the causal association between RA and hypothyroidism/hyperthyroidism. Hypothyroidism confirmed a causal relationship with RA when employed as an exposure variable, whereas no such relationship was found between hyperthyroidism and RA.

## 1 Introduction

Rheumatoid arthritis (RA) is a common autoimmune disease characterized by persistent joint pain and the degradation of joint cartilage and bone. Moreover, it causes varying degrees of harm to various extra-articular systems. In the general population, the prevalence of RA ranges from 0.5% to 1%, with a higher incidence in women than in men (1). RA leads to functional impairment, reduced work capacity, and a lower quality of life, substantially burdening individuals and society (2, 3). Furthermore, RA is associated with a significantly higher mortality rate than the general population, with approximately 40% of patients with RA succumbing to cardiovascular disease (4, 5). Thyroid dysfunction, encompassing hyperthyroidism, hypothyroidism, subclinical hyperthyroidism, and subclinical hypothyroidism, is a common endocrine disorder diagnosed primarily through biochemical indicators such as thyroid stimulating hormone (TSH), triiodothyronine (T3), thyroxine (T4), free triiodothyronine (FT3), and free thyroxine (FT4). The prevalence of hyperthyroidism ranges from 0.2% to 1.3% (6, 7), while that of hypothyroidism varies from 0.2 to 5.3% (6, 8). Hyperthyroidism and hypothyroidism could impact various bodily systems, including the integumentary, muscular, skeletal, cardiovascular, nervous, digestive, endocrine, and circulatory systems. While most studies have suggested a link between RA and thyroid dysfunction (9), one study found no significant difference in the incidence and prevalence of hypothyroidism between patients with and without RA (10).

Therefore, the potential causal relationship between RA and hypothyroidism/hyperthyroidism requires further investigation. Most of our insights into the relationship between hypothyroidism/hyperthyroidism and RA are derived from observational studies, which are susceptible to reverse causality, selective bias, and confounding variables. Therefore, further research employing innovative methodologies is warranted.

Mendelian randomization (MR) is one such technique that utilizes genetic variation as an instrumental variable to assess causal relationships between exposures and specific outcomes (11).

## 2 Method

### 2.1 Data sources

We acquired summary statistics for RA from the MRCIEU GWAS database available at https://gwas.mrcieu.ac.uk/. The pooled GWAS data, involving individuals of European ancestry, comprised a population of 58,284 for subsequent analysis (GWAS ID: ieu-a-832), with 14,361 cases and 42,923 control participants (12). We obtained summary data from the publicly available FinnGen Biobank for the GWAS datasets associated with hypothyroidism and hyperthyroidism. The dataset for hypothyroidism (GWAS ID: finn-b-E4_HYTHYNAS) comprised 26,306 cases and 187,684 controls, while hyperthyroidism (GWAS ID: finn-b-AUTOIMMUNE_HYPERTHYROIDISM) comprised 962 cases and 172,976 controls. In these datasets, hypothyroidism was defined as “Hypothyroidism, other/unspecified,” and hyperthyroidism as “Autoimmune hyperthyroidism.” Data regarding smoking quantity were extracted from the GWAS and Sequencing Consortium of Alcohol and Nicotine use (GSCAN) (13) (cigarettes per day: GWAS ID: ieu-b-25, sample size: 337,334). Summary-level data for alcohol intake frequency were obtained from the UK BioBank (GWAS ID: ukb-a-25, sample size: 336,965). Genetic instruments associated with BMI were sourced from a previously published GWAS study (14). We obtained these data from two consortia: the Genetic Investigation of Anthropometric Traits (GIANT) consortium and the Genetic Epidemiology of Adult Health and Aging Study (GERA) consortium (GWAS ID: ebi-a-GCST006368, sample size: 315,347).

To minimize bias due to population stratification, we focused exclusively on individuals of European ancestry. All datasets are available for download from the IEU GWAS database at this link: https://gwas.mrcieu.ac.uk/datasets/.

A detailed description of the data source is provided in **Supplementary Table 1**.

Since all the data were derived from publicly accessible studies, our research did not require patient consent or ethical clearance.

### 2.2 Study design

Our study design comprised two primary steps. First, we performed a two-sample univariate analysis, using RA as the exposure variable, and examined its association with hypothyroidism/hyperthyroidism as the respective outcomes. Subsequently, we performed another two-sample univariate analysis, using hypothyroidism/hyperthyroidism as the exposure variables, and assessed their relationships with RA as the outcomes. In a second step, to ensure that any causal effects were not influenced by factors such as BMI, smoking quantity, and alcohol intake frequency, a multivariate analysis was conducted to account for the impact of these variables.

### 2.3 Instrumental variable selection

**Figure 1:**
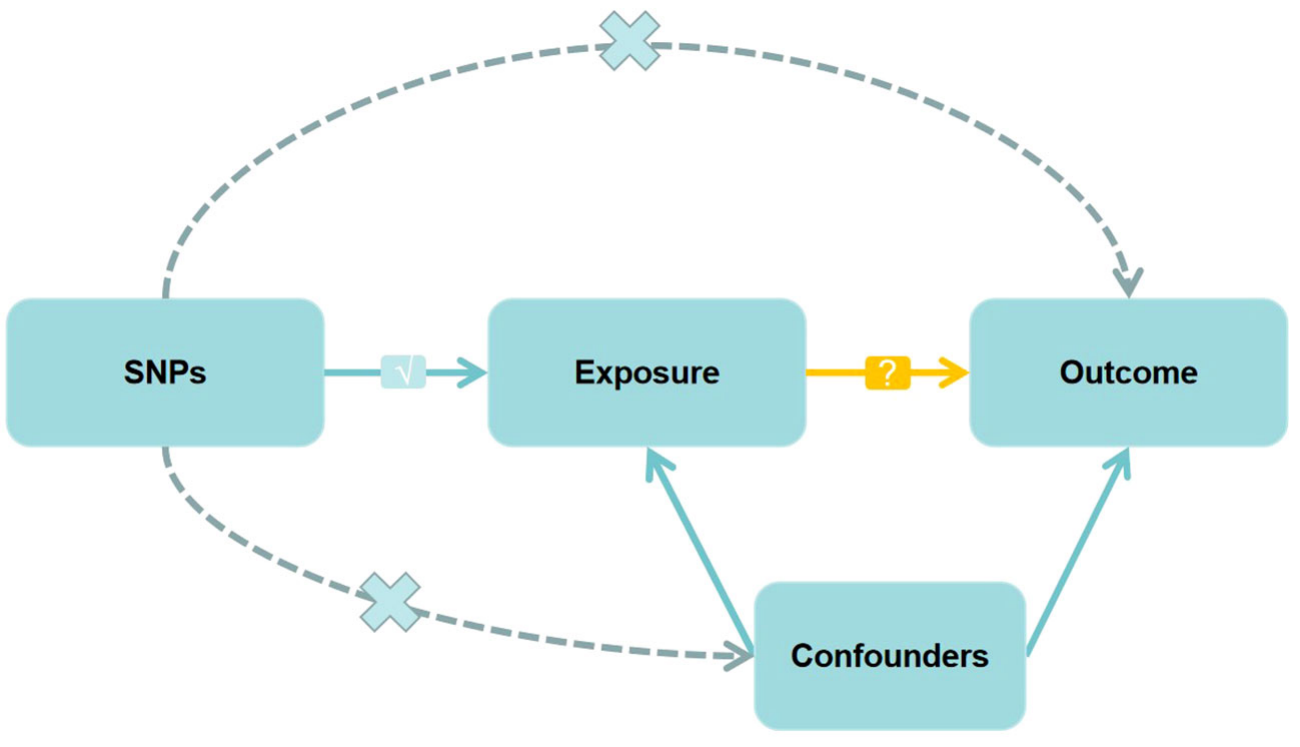
Directed acyclic graph of the Mendelian randomization (MR) framework investigating the causal relationship between exposure and outcome. The ‘×’ means that genetic variants are not associated with confounders or cannot be directly involved in outcome but via the exposure pathway. The ‘√’ means that genetic variants are highly correlated with exposure. SNPs, single-nucleotide polymorphisms.

In MR studies, genetic variants frequently serve as instrumental variables (IVs). Three critical assumptions must be met to obtain reliable casual estimates in MR studies: 1) IVs should exhibit a strong association with the exposure; 2) IVs should not be associated with any potential confounders that might affect the relationship between the exposure and outcome; 3) IVs should exclusively influence the outcome through the exposure (15, 16). **Figure 1** depicts a detailed description.

Firstly, we identified independent single nucleotide polymorphisms (SNPs) significantly associated with the exposure (P<5×10^-8^). We conducted SNP clustering with a window size of 10,000 kb and an R2<0.001 threshold to remove linkage disequilibrium (LD). Subsequently, we checked all the exposure-related SNPs in the PhenoScanner database (http://www.phenoscanner.medschl.cam.ac.uk/) to identify any SNPs associated with potential confounders (BMI, smoking, alcohol consumption) and the outcome (P<5×10^-8^). We extracted SNP effects from the outcome GWAS dataset and harmonized the impact of the exposure and outcome. We excluded palindromic SNPs with ambiguous results (EAF >0.42). We employed the MR-Pleiotropy Residual Sum and Outlier (MR-PRESSO) method to identify and remove potential outliers (17). Finally, we assessed instrumental strength using the F-statistic ( $F=\frac{N-k-1}{k}\times \frac{R^{2}}{1-R^{2}}$ ), following the procedures outlined by Burgess and Thompson (18).

## 3 Mendelian randomization analyses

For our MR analyses, we employed R (version 4.2.2) packages, namely “TwoSampleMR” (version 0.5.6) and “MR-PRESSO” (version 1.0).

We assessed the association between hypothyroidism/hyperthyroidism and RA using three methods: inverse variance weighted (IVW), weighted median (WM), and Mendelian randomization-Egger (MR-Egger) methods. IVW method offers consistent estimates when all genetic variants are valid IVs (19). Conversely, MR-Egger regression provides consistent estimates, notably when all considered genetic variants are incorrect IVs. The weighted median approach generates consistent appraisals, requiring at least half of the weights to be derived from accurate IVs (20). Our primary result was based on the IVW method, while MR-Egger and WM approaches were used to assess the reliability and stability of the results.

We used the MR-Egger intercept test to detect horizontal pleiotropy, with a P-intercept >0.05 indicating the absence of such pleiotropy. We further employed the IVW method and Egger regression to evaluate heterogeneity, with P<0.05 indicating its presence. Cochran’s Q statistic was used to assess heterogeneity (21). Moreover, we conducted a leave-one-out analysis to investigate whether a single SNP was driving the causal association.

### 3.1 Univariable MR estimates

A rigorous screening process included removing specific SNPs, including rs6679677, rs13426947, rs3087243, rs34046593, rs2561477, rs2844456, rs6936656, rs1571878, rs12764378, rs706778, rs8032939, and rs34536443 for their associations with confounders or outcomes. Furthermore, rs1042169, rs13330176, rs225433, rs2661798, rs3799963, and rs4452313 were excluded as they failed to harmonize. Subsequently, we employed 26 valid IVs for MR estimation of RA’s impact on hypothyroidism/hyperthyroidism (**Supplementary Tables 2**, **3**). For the MR estimation of hypothyroidism’s impact on RA, we considered and utilized 31 valid IVs (**Supplementary Table 5**), following the removal of specific SNPs (rs6679677, rs4853458, rs11571297, rs932036, rs9277542, rs707937, rs7902146, rs4409785, and rs7310615) due to their associations with confounders or outcomes, and rs9265890, which failed to harmonize. We considered and utilized three valid IVs for the MR estimation of hyperthyroidism’s impact on RA (**Supplementary Table 5**). These were retained after excluding specific SNPs (rs6679677 and rs9275576) due to their associations with confounders or outcomes and rs9265890, which failed to harmonize.

**Figure 2:**
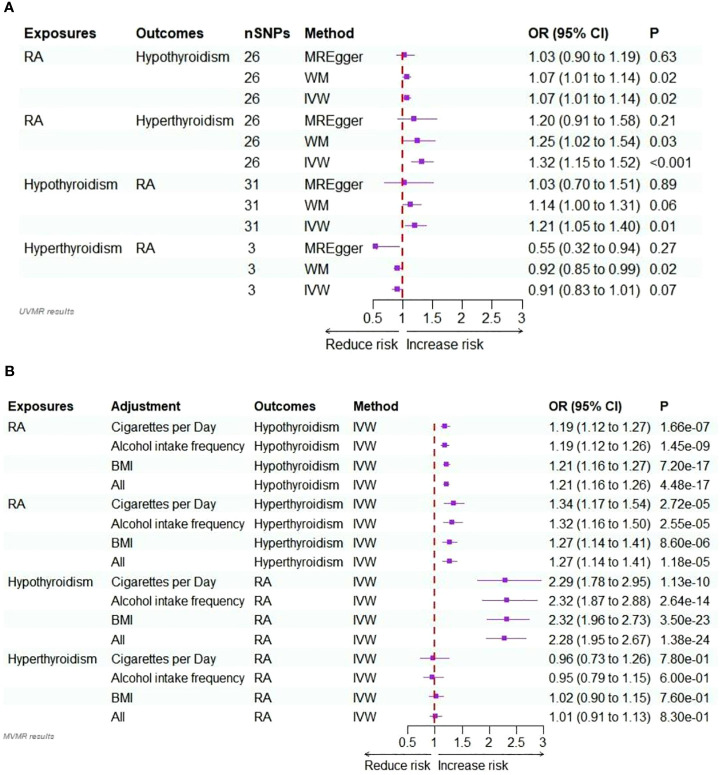
**(A)** Forest plots of the bidirectional two-sample univariable Mendelian randomization analysis of the relationship for RA on hypothyroidism/hyperthyroidism, and hypothyroidism/hyperthyroidism on RA. **(B)** Forest plots of the bidirectional two-sample multivariable Mendelian randomization analysis of the relationship for RA on hypothyroidism/hyperthyroidism, and hypothyroidism/hyperthyroidism on RA.

In the initial step of the univariable MR analysis, we found a significant causal effect of RA on hypothyroidism (IVW: odds ratio [OR] = 1.07, 95% confidence interval [CI] = 1.01–1.14, P = 0.02). The results obtained from the WM method aligned with those from the IVW method (P = 0.02). However, the MR-Egger regression method indicated a similar causal effect direction; however, they did not reach significance (P = 0.63). A significant causal effect of RA on hyperthyroidism was observed (IVW OR = 1.32, 95% CI = 1.15–1.52, P< 0.001). The WM method results were consistent with the IVW method (P = 0.03); however, the MR-Egger regression method results were insignificant (P = 0.21). Conversely, in the inverse analysis, hypothyroidism exhibited a significant causal effect on RA (IVW OR = 1.21, 95% CI = 1.05–1.40, P = 0.01). However, neither reached significance, while the WM method (P = 0.06) and MR-Egger regression (P = 0.89) indicated causal effects in the same direction. However, there was no estimated causal effect of hyperthyroidism on RA in the IVW (P = 0.07) and MR-Egger regression methods (P = 0.27). Although, the WM method (P = 0.02) showed a causal effect of hyperthyroidism on RA, the focus was on the results of the IVW method, as shown in **Table 1** and **Figure 2A**. All instrumental variables used in this study exhibited F-statistic values exceeding 10, indicating the robustness of the selected IVs (**Supplementary Tables 2**-**5**).

**Table 1:** MR Results of RA on Risk of hypothyroidism/hyperthyroidism, and hypothyroidism/hyperthyroidism on Risk of RA.

Exposures	Outcomes	nSNPs	Method	OR (95%CI)	P
RA	Hypothyroidism	26	MR-Egger	1.03(0.90-1.19)	0.63
			WM	1.07(1.01-1.14)	0.02
			IVW	1.07(1.01-1.14)	0.02
RA	Hyperthyroidism	26	MR-Egger	1.20(0.91-1.58)	0.21
			WM	1.25(1.02-1.54)	0.03
			IVW	1.32(1.15-1.52)	<0.001
Hypothyroidism	RA	31	MR-Egger	1.03(0.70-1.51)	0.89
			WM	1.14(1.00-1.31)	0.06
			IVW	1.21(1.05-1.40)	0.01
Hyperthyroidism	RA	3	MR-Egger	0.55(0.32-0.94)	0.27
			WM	0.92(0.85-0.99)	0.02
			IVW	0.91(0.83-1.01)	0.07

RA, Rheumatoid arthritis; IVW, inverse variance weighted; WM, weighted median; nSNPs, number of SNPs used in MR; OR, odds ratio; CI, confidence interval; a Statistically significant (p< 0.05).

In the sensitivity analysis, Cochran’s Q test (P<0.05) revealed heterogeneity in the valid IVs used to estimate the effect of RA on hypothyroidism and hypothyroidism on RA. Therefore, a random effects model was employed. Conversely, the valid IVs used to assess the impact of RA on hyperthyroidism and hyperthyroidism on RA did not exhibit heterogeneity in Cochran’s Q test (P >0.05). Furthermore, there was no evidence of horizontal pleiotropy (P >0.05 for MR-Egger intercept) in any of the univariate MR analyses (**Supplementary Table 6**). The leave-one-out analysis results (**Supplementary Figure 3**) indicated no individual SNP significantly affected the causal effects. Scatter and funnel plots are presented in **Supplementary Figures 1** and **2**.

### 3.2 Multivariable MR estimates

In multivariable MR analysis, we adjusted individually for smoking quantity, alcohol intake frequency, and BMI; strong evidence supported a direct causal effect of RA on the risk of hypothyroidism. This was observed when adjusting for smoking quantity (IVW: OR = 1.19, P<0.001), alcohol intake frequency (IVW: OR = 1.19, P<0.001), and BMI (IVW: OR = 1.21, P<0.001). In multivariable MR analysis jointly adjusted for these factors, the effect size of the association slightly increased (IVW: OR = 1.21, P<0.001).

Similarly, in multivariable MR analysis, when individually adjusting for smoking quantity, alcohol intake frequency, and BMI, strong evidence supported a direct causal effect of RA on the risk of hyperthyroidism. This was observed when adjusting for smoking quantity (IVW: OR = 1.34, P<0.001), alcohol intake frequency (IVW: OR = 1.32, P<0.001), and BMI (IVW: OR = 1.27, P<0.001). In multivariable MR analysis jointly adjusted for these factors, the effect size of the association was slightly reduced (IVW: OR = 1.27, P<0.001).

In the multivariable MR analysis, where we individually adjusted for smoking quantity, alcohol intake frequency, and BMI, there was compelling evidence of a direct causal effect of hypothyroidism on the risk of RA. Specifically, when adjusted for smoking quantity, the IVW OR was 2.29 with P<0.001. Similarly, the IVW OR was 2.32 (P<0.001) when adjusted for alcohol intake frequency and 2.32 (P<0.001) when adjusted for BMI. The association remained strong even in the multivariable MR analysis, simultaneously adjusted for all these factors (IVW: OR = 2.28, P<0.001).

However, there was no discernible evidence of a causal effect of hyperthyroidism on the risk of RA in the multivariate MR analyses, whether adjusted for smoking quantity, alcohol intake frequency, and BMI individually or collectively (P >0.05). Detailed results are provided in **Supplementary Table 7**, **Figure 2B**.

## 4 Discussion

In our study, we performed bidirectional two-sample univariable and multivariable MR analyses to investigate the potential causal relationship between RA and hypothyroidism/hyperthyroidism. The results of the univariable analysis indicated a causal relationship between RA and hypothyroidism/hyperthyroidism. However, when we considered hypothyroidism/hyperthyroidism as exposure factors, we only identified a causal relationship between hypothyroidism and RA and not between hyperthyroidism and RA. In the multivariate MR analyses, after adjusting for smoking quantity, alcohol intake frequency, and BMI, the causal association between RA and hypothyroidism/hyperthyroidism and that between hypothyroidism and RA remained robust.

RA is an autoimmune disease characterized by symmetrical synovial inflammation resulting from a complex interplay of genetic and environmental factors that disrupt immune tolerance. Extra-articular clinical manifestations occur in approximately 40% of patients with RA (22). Thyroid dysfunction represents one of the most common chronic endocrine disorders. Patients with thyroid dysfunction frequently exhibit clinical manifestations similar to those seen in patients with RA, such as fatigue, muscle weakness, joint pain, and swelling. Furthermore, both conditions share similar pathogenic mechanisms involving autoimmune, inflammatory, genetic, and environmental factors. This study focuses explicitly on hyperthyroidism and hypothyroidism in thyroid dysfunction. Thyroid dysfunction, with or without autoimmune thyroid disease (AITD), is observed in 6% to 33.8% of patients with RA (23). The results of a meta-analysis highlight an increased risk of thyroid dysfunction in patients with RA, with a more significant association with hypothyroidism (OR= 2.25, 95% CI=1.78–2.84) than hyperthyroidism (OR 1.65, 95% CI 1.24–2.19), consistent with our findings (9). The predominant causes of hyperthyroidism and hypothyroidism are Graves’ disease and Hashimoto’s thyroiditis, respectively. In a study involving 2791 cases of Graves’ disease and 495 cases of Hashimoto’s thyroiditis, 3.15% of individuals with Graves’ disease and 4.24% of individuals with Hashimoto’s thyroiditis had RA (24). Furthermore, research by Nisihara et al. revealed positive antinuclear antibodies (ANA) in 17.5% of patients with AITD (excluding rheumatic diseases), with rheumatoid factor (RF) detected in 7.7% of such patients (25). In a study by Elnady et al., significant differences were observed with ANA (50.8%), RF (34.4%), and anti-cyclic citrullinated peptide (anti-CCP) (19.7%) (26). Conversely, in a cohort study of 800 patients with RA, 9.8% had AITD, 37.8% tested positive for anti-thyroid peroxidase (TPO) antibodies, and 20.8% were positive for anti-thyroglobulin (TG) antibodies (27). This evidence suggests an association between RA and hypothyroidism/hyperthyroidism, warranting further investigation into the underlying mechanisms that link these conditions.

Autoimmune thyroid disease significantly contributes to thyroid dysfunction. Shared physiopathologic mechanisms exist among autoimmune diseases (AD), including RA and AITD (28, 29). These conditions share common susceptibility genes and environmental factors, which could account for the higher incidence of autoimmune diseases in affected individuals. Some susceptibility genes associated with the development of RA include HLA-DRB1, PTPN22, AFF3, CD28, CD40, CTLA4, IL2RA, IL2, IL21, PRKCQ, STAT4, TAGAP, REL, TNFAIP3, TRAF1, BLK, CCL21, FCGR2A, PADI4, and PRDM1 (30). Furthermore, susceptibility genes for AITD include TSHR, TG, HLA, CTLA4, PTPN22, CD40, CD25, ARID 5B, BT61, FCRL3, IL2RA, and FOXP3 (31). Among these, PTPN22, CTLA4, HLA_DRB1, FCRL3, and IL2RA are common susceptibility genes between both conditions, while CD40 is exclusively associated with Graves’ disease and RA (32). Regarding environmental factors, smoking significantly increases the risk of RA (33) and Graves’ disease (34). Surprisingly, smoking has been found to reduce the risk of hypothyroidism, although this protective effect diminishes in the years following smoking cessation (35–37). Alcohol intake frequency is protective against RA (38, 39). Meanwhile, Carlé demonstrated that moderate alcohol intake frequency has diminished the risk of developing Hashimoto’s thyroiditis and Graves’ disease (40). Furthermore, gut flora dysbiosis has been proposed as an essential environmental factor in developing RA and hypothyroidism/hyperthyroidism (41–43). Chen et al. found that anti-tumor necrosis factor treatment (anti-TNFα) in mice reduced the expression of pro-inflammatory cytokines in the thyroid gland, thereby reducing inflammation (44). Hennie et al. showed that in individuals with RA and hypothyroidism, anti-TNFα therapy was associated with improved thyroid function (45). Moreover, patients receiving anti-TNFα treatment had a lower incidence of thyroid disease than those who did not (46). Elevated levels of interleukin-1 (IL-1) and IL-6 have been associated with the severity of RA and joint damage (47, 48). In summary, our study associates RA with hypothyroidism/hyperthyroidism, confirming that RA increases the risk of hypothyroidism/hyperthyroidism, and conversely, hypothyroidism elevates the risk of RA. However, we did not identify a causal relationship between hyperthyroidism and the risk of developing RA. We propose that hyperthyroidism might have a protective effect against RA by potentially increasing T regulatory cells, countering the adverse effects of hyperthyroidism on autoimmunity. However, further experimental validation with larger sample sizes is essential.

The strengths of our study include the utilization of bidirectional two-sample univariable and multivariable MR analyses to explore the potential causal relationship between RA and hypothyroidism/hyperthyroidism. Furthermore, using exposure and outcome datasets from distinct consortiums minimizes the impact of sample overlap.

However, this research has several limitations. Firstly, despite employing an MR design and excluding known confounders, the unaccounted potential confounders could still affect the results. Secondly, this research exclusively focused on individuals of European ancestry, which could limit the generalizability of the findings to individuals of other ancestral backgrounds. Therefore, caution is warranted when interpreting the implications of our findings for broader populations. Thirdly, autoimmune diseases generally exhibit a higher susceptibility in women than men. However, due to limitations in the available data from the original GWAS, we could not stratify the study by sex. Furthermore, our respective indicators for smoking and alcohol consumption, namely “ cigarettes per day” and “alcohol intake frequency” provide a limited perspective as they only reflect the quantity and frequency of smoking and drinking without capturing the full biological effects. Comprehensive indicators are available, such as the duration, frequency, and type of smoking, and the description of drinking includes the number of units per drink, the total weekly alcohol consumption, and the type of alcohol consumed. Therefore, further analysis is needed to comprehensively explore the effects of smoking and drinking. Finally, hypothyroidism and hyperthyroidism have complex etiologies with multiple subtypes, and the lack of consideration for these subtypes is a limitation in our MR analysis.

## 5 Conclusion

Our study utilized bidirectional two-sample univariable and multivariable MR analytical techniques to investigate a potential causal relationship between RA and hypothyroidism/hyperthyroidism. We confirmed the causal relationship between RA and hypothyroidism/hyperthyroidism. When hypothyroidism/hyperthyroidism were considered as exposure factors, we identified a causal relationship between hypothyroidism and RA; however, no such association was identified between hyperthyroidism and RA.

## Data availability statement

The original contributions presented in the study are included in the article/**Supplementary Material**. Further inquiries can be directed to the corresponding author.

## Ethics statement

Ethical review and approval were not required for the study on human participants in accordance with the local legislation and institutional requirements. Written informed consent from the participant’s legal guardian/next of kin was not required to participate in this study in accordance with the national legislation and institutional requirements.

## Author contributions

RL: Conceptualization, Data curation, Formal Analysis, Methodology, Writing – original draft. XD: Data curation, Visualization, Writing – original draft. XL: Visualization, Writing – original draft. QL: Data curation, Visualization, Writing – original draft. KZ: Data curation, Visualization, Writing – original draft. DP: Supervision, Writing – review & editing.
