# Supplementary material for: Causal relationship between rheumatoid arthritis and hypothyroidism or hyperthyroidism: a bidirectional two-sample univariable and multivariable Mendelian randomization study
Source: Front Endocrinol (Lausanne). 2023 Nov 29;14:1256208. doi: 10.3389/fendo.2023.1256208 (PMC10716525; doi:10.3389/fendo.2023.1256208)
Supplement: Supplementary file 1 [file DataSheet_1.zip › Supplementary Data Sheet 1.DOCX]

**Supplementary material**

**Supplementary Figure 1. Scatter plots**


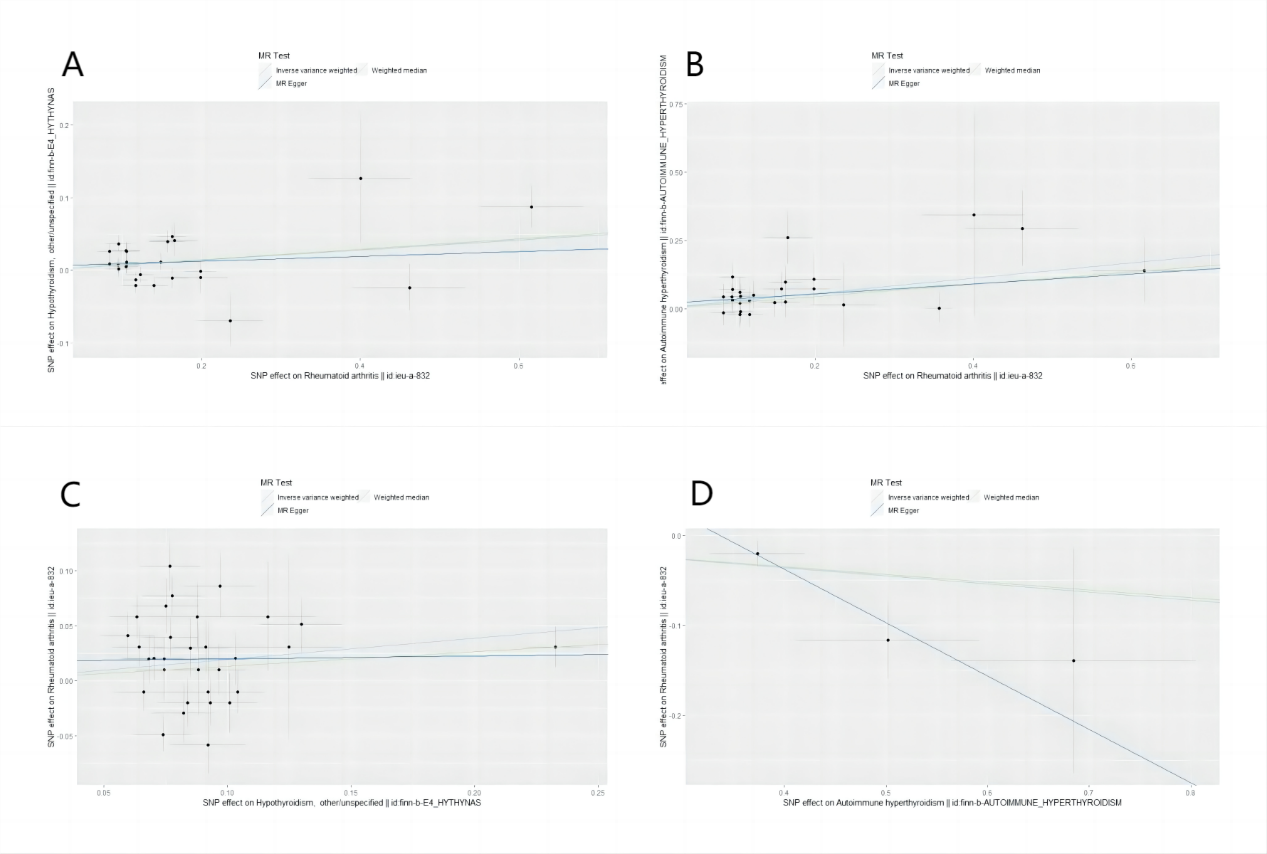


The estimate of intercept can be interpreted as an estimate of the average pleiotropy of all single-nucleotide polymorphisms (SNPs), and the slope coefficient provides an estimate of the bias of the causal effect. (A) RA on Hypothyroidism. (B) RA on Hyperthyroidism. (C) Hypothyroidism on RA. (D) Hyperthyroidism on RA.

**Supplementary Figure 2. Funnel plots**


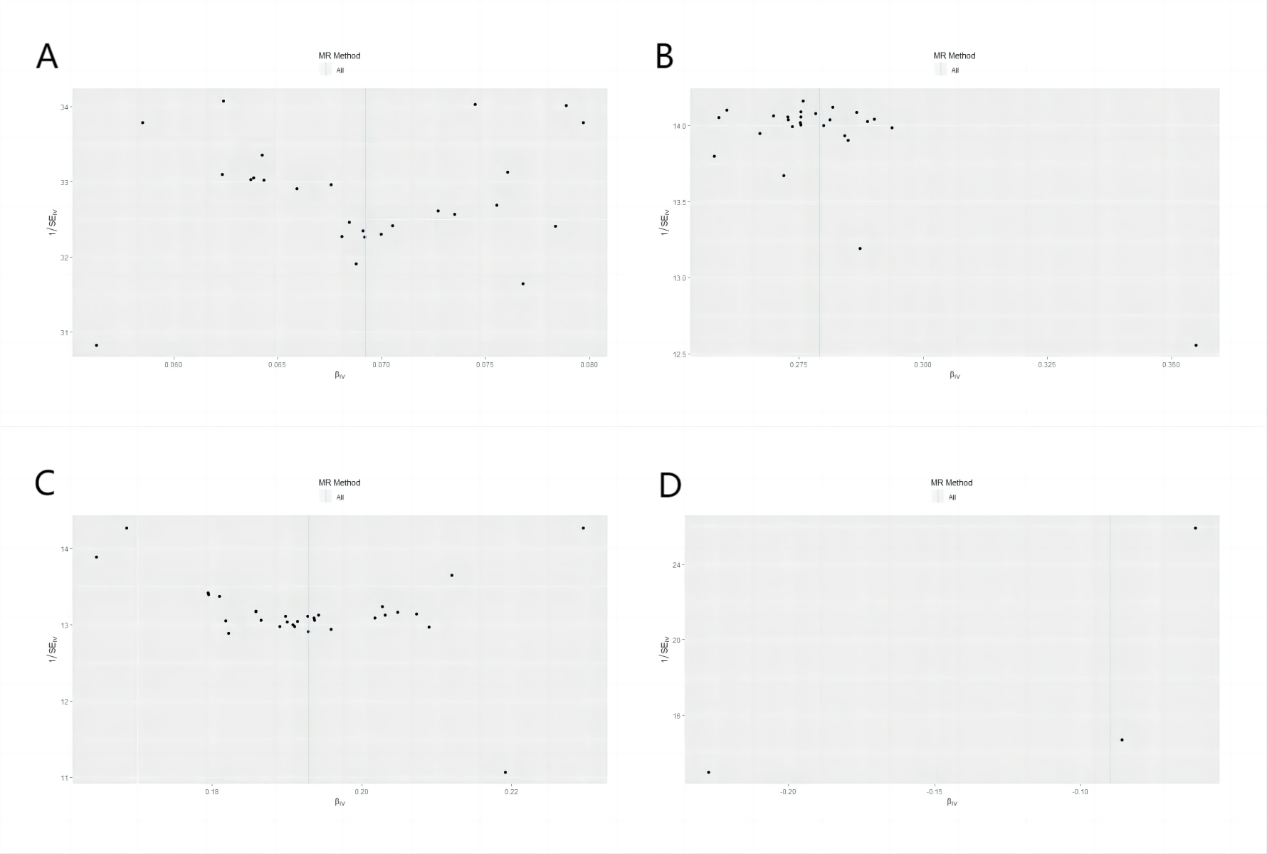


The x-axis represents β, and the y-axis represents 1/SE (standard error). (A) RA on Hypothyroidism. (B) RA on Hyperthyroidism. (C) Hypothyroidism on RA. (D) Hyperthyroidism on RA.

**Supplementary Figure 3. Leave-one-out sensitivity analysis**


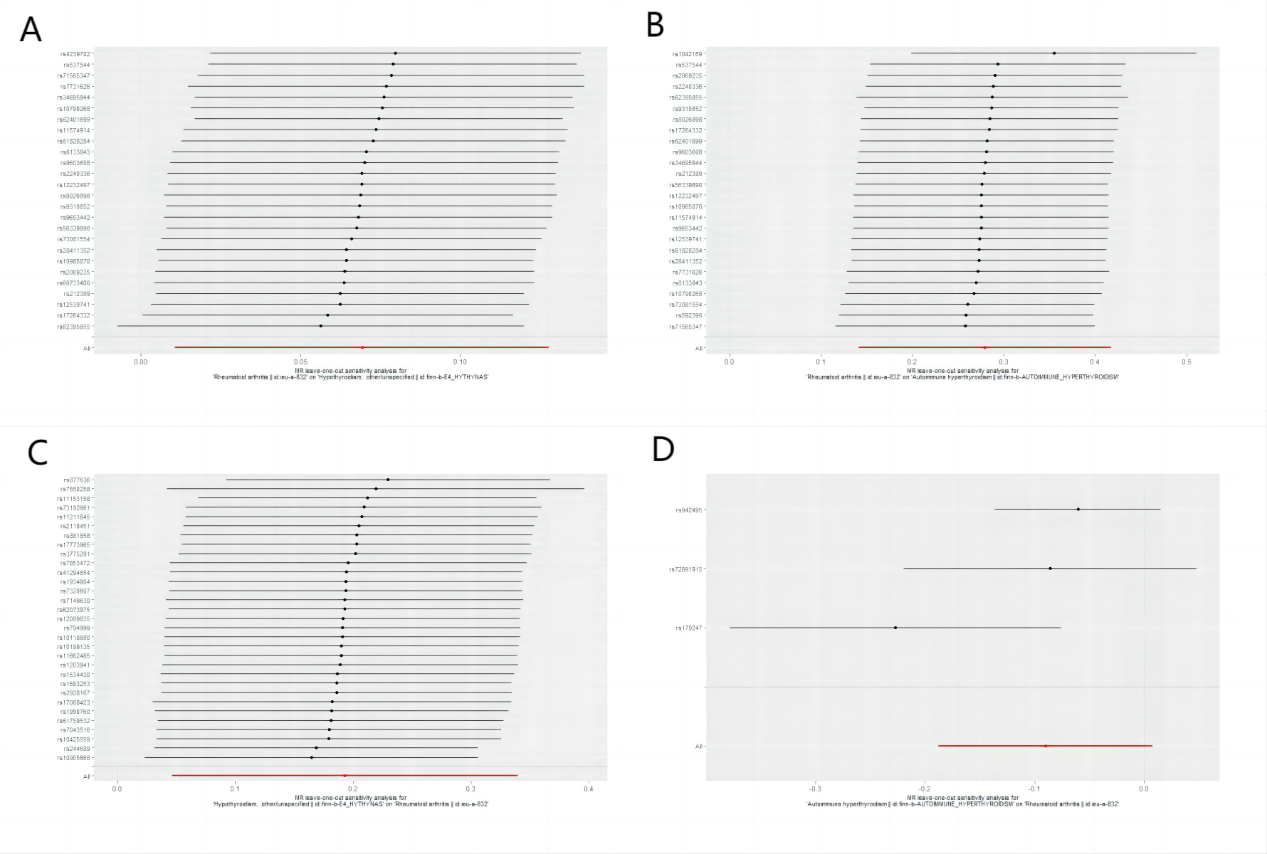


(A) RA on Hypothyroidism. (B) RA on Hyperthyroidism. (C) Hypothyroidism on RA. (D) Hyperthyroidism on RA.
